# Editorial: Teacher responses to bias-based bullying

**DOI:** 10.3389/fpsyg.2025.1592584

**Published:** 2025-04-02

**Authors:** Maria Sapouna, Hildegunn Fandrem, Roy A. Willems

**Affiliations:** ^1^School of Education and Social Sciences, University of the West of Scotland, Paisley, United Kingdom; ^2^Norwegian Centre for Learning Environment and Behavioural Research in Education, University of Stavanger, Stavanger, Norway; ^3^Department of Health Psychology, Faculty of Psychology, Open Universiteit, Heerlen, Netherlands

**Keywords:** bias-based bullying, teachers, bullying, professional ethos, pedagogy of discomfort, racist bullying, racist peer victimization

A significant number of children and young people worldwide are negatively affected by bias-based bullying. Evidence from the broader literature on interpersonal bullying suggests that teachers play a critical role in nurturing a safe and inclusive school environment (Aguirre et al., 2021; Poteat et al., 2019). However, less is known about how teachers intervene and support students in instances of bias-based bullying, which is motivated by prejudice against a person's actual or perceived identity. The aim of this Research Topic was to collate new research that sheds light on teachers' responses to bias-based bullying, barriers to intervention and the most effective strategies for supporting students and cultivating safe and inclusive environments.

In their article, Sieben-Aduful et al. investigated teachers' responses to racist bullying and racist incidents in Dutch primary schools through semi-structured interviews with nine teachers. The study concluded that teachers are reluctant to acknowledge a racist motive behind students' behavior and tend to manage racist bullying as ‘just bullying' therefore ignoring the particularly negative effects that racism can have on young people. The majority of the discussions that teachers led in the classroom were reactive after an incident occurred and focused on cultural diversity rather than racism. The article also found that teachers were hampered by a lack of training on how to effectively respond to racist incidents at school.

In their article, Hay et al. reached surprisingly similar conclusions about how Scottish teachers intervene in cases of racist bullying. Their study analyzed 13 teachers' responses to hypothetical vignettes about racist bullying. Similarly to the findings of Sieben-Aduful et al., this study found that teachers engaged students in discussions about racism in response to the occurrence of incidents as opposed to an embedded curricular approach. In this study too there was reluctance on the part of the teachers to acknowledge the vignettes as racist incidents. Furthermore, a conclusion similar to that of Sieben-Aduful et al. was reached regarding how the lack of training resources such as racial literacy development programs limits teachers' ability to respond effectively to racist incidents.

The article by Bayram Özdemir and Özdemir investigated the relative contributions of teachers' *general efficacy* (i.e., managing disruptive classroom behaviors) and *diversity-related efficacy* (i.e., addressing diversity challenges) to their responses to incidents of ethnic victimization. This quantitative study conducted with a sample of 72 head teachers of 8th-grade students in Sweden found that teachers adopt a diverse range of strategies to address incidents of ethnic victimization, often focusing on comforting the victim. In a hypothetical victimization incident, teachers with a high sense of efficacy for classroom management, rather than efficacy in addressing diversity-related issues, were more likely to indicate that they would contact the parents of both victims and perpetrators and would comfort the victim. According to the authors, these findings highlight the importance of supporting teachers to enhance their efficacy in classroom management to enable them to also respond more effectively to incidents of ethnic victimization. However, the authors also acknowledge that other relevant factors such as the teachers' attitudes toward diversity, their beliefs about the normality of such incidents, and the accuracy of their judgments need to be considered to promote effective responses to bias-based bullying.

In their article, Thomassen et al. used the concept of the *pedagogy of discomfort* to investigate how feelings of discomfort hinder the practice of teachers and preservice teachers in addressing bias-based bullying episodes. The study was based on semi-structured interviews conducted with 14 pre-service and in-service teachers in Norway. In line with previous research (Røthing, 2019), the study concluded that instances of bias-based bullying generated feelings of discomfort that in-service teachers in particular were reluctant to utilize constructively to educate children and young people about sensitive topics. Such feelings of discomfort hindered in-service teachers in particular from acknowledging the racist motive of bullying incidents, a finding that replicates the analyses of Sieben-Aduful et al. and Hay et al. above. Teachers in this study also reported that the daily pressures of classroom management prevented them from paying adequate attention to instances of bias-based bullying. The article concludes that the pedagogy of discomfort may be a useful tool for prevention and intervention in bias-based bullying, in particular for in-service teachers.

The final article by Gutzwiller-Helfenfinger is a conceptual analysis of promoting teachers' *professional ethos*—in short, their moral values and beliefs about their work and the associated tasks and obligations, as manifested in their professional behavior—to contribute to more effective teacher responses to bias-based bullying. The author argues that teachers who display professional ethos are those who show concern by taking action against bias-based bullying and actively promoting an inclusive environment. However, as pointed out by previous articles in this Research Topic, it is important to acknowledge that teachers' professional ethos is embedded in the systemic context of the school and educational system. As the author very poignantly points out “we cannot put the ‘ethos burden' on teachers' shoulders only.”

Indeed, a key message that emerges from all the articles in this Research Topic is that when teachers dismiss bias-based bullying incidents it is often because they lack the knowledge, language and skills to deal with these episodes effectively as has been found in previous research (e.g., McIntyre, 2009). This lack of confidence in addressing bias-based bullying in schools is underpinned by a lack of training and institutional support for embedding an inclusive curriculum. Future research and evidence-based interventions are needed to more fully understand teachers' responses and support them in preventing and reducing the impact of bias-based bullying. Nonetheless, this Research Topic has highlighted a clear need for changes in the professional development of teachers across Europe to ensure that they are well-equipped to address issues of racism, homophobia and transphobia in their classrooms.
